# Molecular Identification of Invasive Non-typeable Group B *Streptococcus* Isolates From Denmark (2015 to 2017)

**DOI:** 10.3389/fcimb.2021.571901

**Published:** 2021-03-29

**Authors:** Hans-Christian Slotved, Kurt Fuursted, Ioanna Drakaki Kavalari, Steen Hoffmann

**Affiliations:** Neisseria and Streptococcus Reference Laboratory, Department of Bacteria, Parasites and Fungi, Statens Serum Institut, Copenhagen, Denmark

**Keywords:** Denmark, capsular genes, genotyping, non-typeable, serotyping, *Streptococcus agalactiae*

## Abstract

The number of invasive *Streptococcus agalactiae* (GBS) non-typeable (NT) isolates in Denmark received since 1999 has in general accounted for 10% of all invasive GBS isolates. We present data on 55 clinical NT isolates based on clinical manifestations, clonal relationship, antimicrobial resistance (AMR) determinants, and virulence factors. The GBS isolates included in this study were phenotypic-based NT obtained from 2015 to 2017, as well as 10 reference isolates. Whole genome sequencing (WGS) was performed on all isolates and the data were analyzed for the presence of both species specific genes, capsular genes (genotype), and other relevant genes. We furthermore compared different procedures for detection of serotype specific capsular genes. Overall we were able to genotype 54 of the 55 isolates. After retesting the isolates a phenotype was detected for 20 (36%) isolates, of which the initial phenotyping problem for 13 isolates was found to be due to a problem with serotype Ia specific antiserum. Thirty-five isolates remained phenotypic non-typeable with a majority of genotype V isolates which do not express a capsular gene. From all the Danish invasive GBS isolates from 2015 to 2017, the 35 NT isolates were all detected in the age group above 21 years with bacteremia. The 35 NT isolates belonged to six different well-known human pathogenic clonal complexes. The CDC recommended sequences for capsule genotyping were the most optimal for serotype prediction, because of the sequence simplicity and clear cutoff values. However we recommend to also use other capsular sequences for the NT isolates, if they cannot be genotyped by the CDC method.

## Introduction

*Streptococcus agalactiae* (group B streptococcus, GBS) is a well-known pathogen primarily causing infections in newborns and the elderly (Brigtsen et al., 2015; Ballard et al., 2016). Identification and surveillance of GBS in humans are therefore essential (Ballard et al., 2016; Sheppard et al., 2016). The GBS are divided into 10 serotypes based on type specific capsular antigens and are designated as Ia, Ib, II, III, IV, V, VI, VII, VIII, and IX (Slotved et al., 2007). In general, isolates are serotyped by phenotypic methods, such as latex agglutination test (latex test) and the precipitation test also known as the Lancefield precipitation test (Slotved and Hoffmann, 2017). However, molecular techniques for genotyping of GBS isolates are increasingly being used, based on PCR assays and whole genome sequencing (WGS) (Streptococcus Laboratory, CDC, https://www.cdc.gov/streplab/groupb-strep/index.html accessed 03-08-2021) (Brigtsen et al., 2015; Sheppard et al., 2016).

It is well-known that a certain percentage of invasive GBS isolates are non-typeable (NT) by phenotypic methods, either because they do not express their capsule or the capsule is hitherto uncharacterized capsule (Rosini et al., 2015; Alhhazmi and Tyrrell, 2018). In recent epidemiological studies NT isolates are generally found to constitute 5–10% of the invasive GBS isolates (Lamagni et al., 2013; Alhhazmi et al., 2016; Björnsdóttir et al., 2016). Since 1999, NT isolates in Denmark have generally accounted for 10% of all invasive GBS isolates received (Lambertsen et al., 2014; Lambertsen et al., 2010), although occasionally the percentage of NT has reached 17% of all invasive GBS cases (Slotved and Hoffmann, 2020).

As only limited data exists, we have characterized a group of phenotypic based GBS NT isolates from 2015 to 2017 as well as 10 reference isolates. The source of isolation for the NT isolates was investigated to find out if they were from a specific group of patients, based on age, sex, and clinical diagnosis. WGS were performed on all isolates, and the data were analyzed for the presence of species specific genes, capsular genes, and other relevant genes. We furthermore compared three different sequence analysis approaches for prediction of serotypes from genome sequence data.

## Materials and Methods

Ten reference strains and 55 clinical isolates non-typeable by phenotypic methods were selected for characterization by WGS (**Supplementary Table 1**). The 10 reference strains represent each one serotype (serotype Ia–IX), and are used as reference strains for GBS at the national Neisseria and Streptococcus Reference (NSR) laboratory, Statens Serum Institut (SSI). Details on the 10 reference isolates are presented in the study by Slotved et al. (2007).

The 55 invasive strains initially reported as non-typeable were isolates from blood or other normally sterile sites from the period 2015 to 2017. The isolates are part of the clinical isolates received at NSR for the years 2015 to 2017, for which details can be found in the study by Slotved and Hoffmann (Slotved and Hoffmann, 2020). The phenotypes of the clinical isolates were identified as described in previous studies (Slotved and Hoffmann, 2017; Slotved and Hoffmann, 2020). Briefly, all isolates were serotyped using GBS latex agglutination test (SSI Diagnostica, Denmark), and if the result was inconclusive, by applying the capillary precipitation method (Lancefield method). If this procedure did not lead to a phenotypic type designation, the isolate was categorized as being non-typable (NT).

### Molecular Sequencing

The genomic DNA of isolates were sequenced by paired-end Illumina sequencing. Genomic DNA was extracted using a DNeasy Blood & Tissue Kit (QIAGEN, Hilden, Germany) and fragment libraries were constructed using a Nextera XT Kit (Illumina, Little Chesterford, UK) followed by 250-bp paired-end sequencing (MiSeqTM; Illumina) according to the manufacturer’s instructions. The paired-end Illumina data were *de novo* assembled using SKESA assembler (Souvorov et al., 2018).

Bioinformatics, including Blast, was performed using the software CLC Main Workbench (Version 8.1, www.qiagenbioinformatics.com).

### The Gene Profiles of the Isolates

GBS species identification for all 65 isolates was performed by detection of 16S rRNA gene (AF015927.1), the housekeeping gene *sodA* (DQ232566.1), and the *cfb* (JQ289578) gene coding for the CAMP factor (Rosa-Fraile and Spellerberg, 2017, Streptococcus Laboratory, CDC, https://www.cdc.gov/streplab/groupb-strep/index.html, access 03-03-2021).

Also other relevant genes were identified: The housekeeping gene *infB* (AJ003164) (Hedegaard et al., 2000; Skov Sørensen et al., 2010), the *hvgA* gene (Alhhazmi et al., 2016) for the surface-anchored adhesion molecule, the gene for the laminin-binding protein [*lmb* (AF062533)], and the gene for group B streptococcal C5a peptidase [*scpB* (SAU56908)] (Shabayek and Spellerberg, 2018).

The presence/absence of a gene was based on a cutoff of 80% coverage and a 95% identity for a positive gene detection.

The genomic sequence data for the 10 reference strains and the 55 clinical isolates are deposited in the Genbank (https://www.ebi.ac.uk/ena, accessed 03-03-2021) (ENA accession no. are PRJEB43628 and PRJEB38759).

### Molecular Identification of Capsular Genes

Sequences from all 65 isolates were blasted against the capsular polysaccharide genes (CPS genes) for all 10 known serotypes by three different sets of described sequences.

Method 1: Ten capsular sequences described by Metcalf et al., (2017). We used the requirements of identity for each sequence described by MMetcalf et al. (2017). This method is also the genotyping procedure for GBS recommended by CDC (https://www.cdc.gov/streplab/groupb-strep/index.html, accessed 03-03-2021).
Method 2: Ten capsular locus sequences described by Kapatai et al. (2017). Identification of serotype was performed according to the presence/absence of genes using a cutoff of 90% coverage and a 95% identity Kapatai et al. (2017).
Method 3: Nine capsular sequences described by Sheppard et al. (2016). Because the sequence for genotype IX was not described in the study, this method was only used for confirming the genotyping results from methods 1 and 2 for the nine described capsular sequences. The threshold for genotyping was 95% sequence identity over 90% of the sequence length (Sheppard et al., 2016).

### Multilocus Sequence Typing (MLST) and Construction of a Phylogenetic Tree

A phylogenetic tree based on single nucleotide polymorphisms (SNPs) analysis of the core genome was performed on the 65 isolates. The core genome was defined by the isolate GBS-ref BIa Bo90 (ATCC, 12400) with an overall core size for this collection of 79.16% (1613548 bp). Identification of SNPs was performed using BWA-mem for mapping and GATK with filtering set to remove positions with less than 10-fold depth and 90% unambiguous variant calls as implemented in NASP against the sequence from isolate GBS-ref BIa Bo90 (ATCC, 12400), which was used as a reference strain in the SNP alignment after removal of duplicated regions using NUCmer (Sahl et al., 2016). The resulting SNP matrix was purged for recombination using Gubbins (Croucher et al., 2015). CLC Main Workbench (Version 8.1, www.qiagenbioinformatics.com) was used to visualize the phylogenetic tree.

### Resistance

The 65 GBS genomes were analyzed for genes conferring resistance to macrolide, lincosamide and streptogramin B, and chloramphenicol using the Resfinder-3.1 (https://cge.cbs.dtu.dk/services/ResFinder/) (80% ID threshold and 60% minimum length settings) (Zankari et al., 2012). Information of the detected genes related to resistance are presented in **Supplementary Table 2**.

Genotypic antibiotic susceptibility profile for penicillin PEN antimicrobial sensitivity in GBS is associated with the penicillin-binding protein2x (PBP2x) (Metcalf et al., 2017). The 55 isolates and the 10 reference strains were analyzed for their PBP signature, based on the protein sequence proposal described by Metcalf et al. (2017), where a PBP2X number determines the level of beta-lactam resistance (**Supplementary Table 2**).

The 55 clinical isolates were tested for antibiotic susceptibility as part of routine laboratory testing previously described by Slotved and Hoffmann (2020). Briefly, all isolates were screened for sensitivity to erythromycin (15 μg discs), clindamycin (2 μg discs), and penicillin G (1 μg discs). D test was performed to detect inducible clindamycin resistance according to the description by EUCAST [EUCAST Clinical Breakpoint table v 5.0 (2015), table v 6.0 (2016), and table v 7.1(2017)] (www.eucast.org/clinical_breakpoints). For non-susceptible isolates the minimum inhibitory concentration (MIC) of erythromycin and clindamycin was determined using Etest^®^ (bioMérieux, Denmark). Antibiotic susceptibility was determined in accordance with the recommendations by EUCAST from, 2015 to, 2017 [EUCAST Clinical Breakpoint table v 5.0 (2015), table v 6.0 (2016), and table v 7.1 (2017)] (www.eucast.org/clinical_breakpoints, accessed 03-03-2021).

Data are presented for erythromycin (ERY), clindamycin (CLI), and penicillin (PEN) (**Supplementary Table 2**).

### Ethical Considerations

The data and samples from patients were collected routinely for national surveillance purposes, therefore no ethical approval or informed consent from patients or guardians were required. The study was approved by the Danish Data Protection Agency (record number, 2007-41-0229). For further details on SSI’s permission to present epidemiological data, see: https://en.ssi.dk/.

## Results

### Characterization of the Clinical Isolates

Initially 55 GBS isolates were categorized as phenotypic non-typeable isolates, however retesting the isolates by phenotypical methods revealed serotype identification of 20 isolates (**Supplementary Table 1**. Of the 55 phenotypic non-typeable isolates, 27 were from 2017, 18 from 2016, and 10 from 2015.

The remaining 35 phenotypic NT isolates were all from blood cultures. Of the 35 patients, 21 (60%) were male. The median age was 70 years, with an interquartile range of 65–95 years and a range of 22–95 years. There were no NT isolates detected from early-onset disease (EOD) (age 0–6 days) and late-onset disease (LOD) (age 7–90 days) or from age groups below 22 years of age (**Table 1**).

**Table 1:** Clinical characteristics of 35 phenotypic non-typeable isolates from blood samples.

Isolate number	Age	Sex	Genotype	MLST (ST)	Clonal Complex (CC)	*scpB* (SAU56908)	*lmb* (AF062533)
Strains from 2017							
15-2017	85	Male	VIII	1	1	Present	Present
182-2017	73	Male	Ib	10	6-8-10	Present	Present
286-2017	68	Female	V	1	1	Absent	Absent
292-2017	21	Female	Ia	88	23	Present	Present
306-2017	79	Female	Absent	1-1-1-1-2-2-unknown	1	Present	Present
365-2017	72	Male	Ia	498	23	Present	Present
405-2017	44	Female	Ib	9	6-8-10	Present	Present
466-2017	73	Male	Ib	10	6-8-10	Present	Present
521-2017	70	Male	II	12	6-8-10	Present	Present
527-2017	70	Male	Ib	1-4-1-3-3-2-unknown	6-8-10	Present	Present
538-2017	94	Male	IV	196	196	Present	Present
705-2017	90	Male	IX	130	130	Present	Present
712-2017	48	Male	V	1	1	Absent	Present
Strains from 2016							
129-2016	65	Female	Ib	10	6-8-10	Present	Present
145-2016	87	Female	IV	196	196	Present	Present
168-2016	69	Female	V	1	1	Absent	Present
195-2016	27	Male	III	19	19	Absent	Absent
196-2016	72	Female	V	1	1	Absent	Present
197-2016	89	Male	Ia	144	23	Present	Present
228-2016	65	Male	V	1	1	Absent	Present
246-2016	85	Male	V	1	1	Absent	Present
267-2016	41	Male	Ib	12	6-8-10	Present	Present
319-2016	69	Female	V	1	1	Absent	Present
348-2016	68	Male	Ia	88	23	Present	Present
465-2016	78	Male	Ia^a^	4	1	Present	Present
578-2016	70	Female	V	1	1	Absent	Present
653-2016	84	Female	II	10	6-8-10	Present	Present
Strains from 2015							
21-2015	74	Female	IX	130	130	Present	Present
59-2015	50	Male	V	1	1	Absent	Present
96-2015	77	Male	III	19	19	Present	Present
171-2015	55	Male	V	19	19	Present	Present
360-2015	73	Male	II	28	19	Present	Present
362-2015	51	Male	V	7	6-8-10	Present	Present
480-2015	70	Female	V	1	1	Absent	Present
600-2015	69	Female	V	1	1	Absent	Present

a Method 1 did not detect any genotype.

### Identification of the Genotype

Method 1 (Metcalf et al., 2017) detected capsular genes in 52 of the 55 clinical isolates, while method 2 (Kapatai et al., 2017)detected capsular genes in 54 of the isolates.

Phenotypic retesting of all isolates with reagents corresponding to the detected genotypes provided a corresponding phenotype in 20 isolates, while 35 isolates still were determined as NT isolates. Among 10 NT isolates from, 2015, two isolates were found to express their capsule (one serotype Ia and one serotype V), and for 2016 four isolates of the initial 18 NT isolates were found to express their capsule (one serotype Ia, one serotype V, one serotype VII, and one serotype IX). Regarding 2017, 14 of the initial 27 NT isolates were found to express their capsule, 13 were serotype Ia and one was serotype IX.

Regarding those isolates from 2017 that were phenotypic NT but genotypic identified as serotype Ia, repeated phenotypic typing with a different batch of type specific antiserum identified the majority (13 of 27 isolates, 48%) as type Ia. The identification of the majority of serotype Ia isolates from 2017 as NT was therefore mainly due to antiserum problems and not a change in capsular expression.

Four isolates with capsular locus sequences showed discrepancies between the three methods.

**Figure 1:**
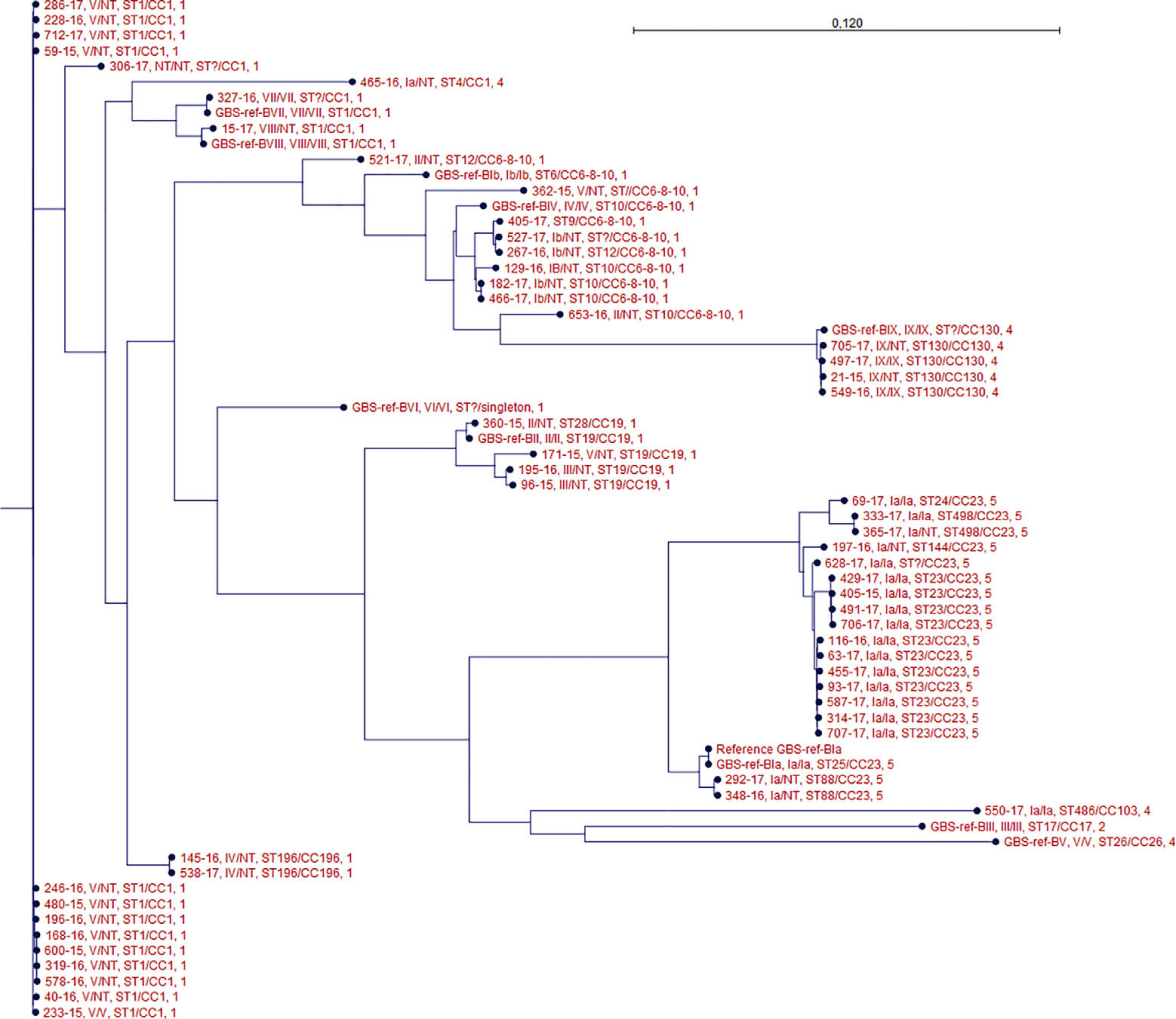
Phylogenetic tree of all isolates. For each isolate data are presented for the genotype/phenotype, MLST type/clonal complex, and PBP profile. Isolate GBS-ref-Bia (Bo90 - ATCC, 12400) was used as a reference strain in the SNP alignment.

One isolate (306-2017) did not present capsular genes using any of methods 1, 2, and 3 or expressed any known phenotypic capsule. The isolate presented an ST type with one unknown allele, however it belonged to CC 1. It did not show close clonal relation to any of the other isolates in the phylogenetic tree (**Figure 1**).

Method 1 did not detect any capsular genes in isolate 116-2016, while methods 2 and 3 detected capsular genes for serotype Ia, confirmed by repeated phenotypic test. The isolate clustered together with other serotype Ia isolates (**Figure 1**).

Method 2 detected serotype Ia capsular genes in NT isolate (465-2016), while this was not the case with method 1. Method 3 also detected serotype Ia capsular genes for these two isolates. No phenotype could be detected. The isolate was ST type 4 and belonged to CC 1. It did not show close clonal relation to any of the other isolates in the phylogenetic tree (**Figure 1**).

Methods 1 and 3 detected serotype III capsular gene for isolate (96-2015) while method 2 detected serotype Ia capsular genes. No phenotype could be detected. The isolate was ST type 19 and belonged to CC 19. Isolate 96-2015 showed close clonal relationship to isolate 195-2016 which was another NT defined isolate in which capsular gene for serotype III was detected by all three methods (**Figure 1**).

Data on the four isolates are presented in **Supplementary Table 3**.

### Isolates Phenotypic Confirmed as Being NT

The sequences from the 35 isolates phenotypic confirmed as non-typeable showed capsular genes for eight of 10 described serotypes excluding serotype VI and VII. The genotyping showed a high predominance of serotype V (13 isolates) followed by serotype Ia (6 isolates), Ib (6 isolates). One isolate (306-2017) did not present capsular genes (**Supplementary Tables 3**, **4**).

### Characterization of Selected Genes

The 16sRNA gene specific for GBS, the CAMP factor gene *cfb*, and the housekeeping genes *infB* and *sodA* were detected in all 55 isolates.

The *hvgA* gene was only observed in reference strain (M781) for serotype III, while this gene was not detected in any of the clinical isolates. All isolates had the *scpB* gene, except the reference strain (serotype V, 1169 NT1), one serotype Ia isolate, two serotype V isolates, and 12 serotype NT isolates. The *lmb* gene appeared in all strains except five: one reference strain (serotype V, 1169 NT1), two NT isolates (286-2017, 195-2016), one serotype Ia isolate (550-2017), and one serotype V isolate (40-2016). These five isolates also did not have the *scpB* gene. For additional information on the selected genes, see **Supplementary Table 1**.

### Phylogenetic Description

The 35 isolates verified as NT belonged to six different clonal complexes (**Table 1**) among which CC1 was the most frequent one including 14 the isolates, followed by CC6-8-10 (9 isolates), CC19 (four isolates), CC23 (4 isolates), CC130 (two isolates), and CC196 (two isolates). There were no singletons. One isolate belonging to CC1 and one isolate belonging to CC6-8-10 both showed an unknown locus.

MLST sequence types correlated to clade relationships depicted in the core SNP phylogeny (**Figure 1**).

The genotype of the phenotypic non-typeable isolates and the genotype of the phenotypeable isolates were generally clustered together (**Figure 1**).

### Penicillin Susceptibility

All 55 clinical isolates were phenotypically susceptible to penicillin (Slotved and Hoffmann, 2020). The PBP2x protein sequence type for all 65 isolates belonged to four different PBP2x types, type 1, 2, 4, and 5 as defined by Metcalf et al., 2017). Comparing the PBP2x profiles with phenotypic penicillin susceptibility (**Supplementary Table 2**showed full agreement according to the prediction model for PBP2x created by Metcalf et al. (Metcalf et al., 2017).

### Erythromycin and Clindamycin Susceptibility

All 55 clinical isolates and the 10 reference strains harbored the *mre(A)*(U92073) gene, and 9 reference and 44 isolates harbored only this gene. Among these 44 isolates only one (15-2017) showed non-susceptibility against both erythromycin and clindamycin (**Table 2**).

**Table 2:** Association of genes related to antimicrobial susceptibility in 55 clinical GBS isolates from 2015 to 2017.

Resistance genes for erythromycin and clindamycin	No. of strains	Erythromycin R <21mm (15 µg) and MIC R >0.5^a^	Clindamycin R <17mm (2 µg) and MIC R >0.5^a^
None	0	0	0
mre(A)(U92073)	44	1^b^	1^b^
mre(A)(U92073)Isa(C)(HM990671)	2	0	2
mre(A)(U92073)erm(B)(U86375)	4	4^c^	4^c^
mre(A)(U92073)mef(A)(U83667) msr(D)(AF274302)	4	4^d^	0
mre(A)(U92073) erm(A)(AF002716) mef(A)(AJ971089) msr(D)(AF274302)	1	1^e^	1^e^

^a^The isolates that were resistant according to the inhibition zone diameter found with the disc diffusion test were further tested by determination of MIC using Etest^®^ (bioMérieux, Denmark) in accordance with the recommendations by EUCAST from, 2015 to, 2017 [EUCAST Clinical Breakpoint table v 5.0 (2015), table v 6.0 (2016), and table v 7.1 (2017)] (www.eucast.org/clinical_breakpoints, accessed 03-03-2021), ^b^identical isolate, ^c^identical isolates, ^d^identical isolates, ^e^identical isolate.

Detailed data for each isolate are presented in **Supplementary Table 2**.

Two isolates harbored both the *mre(A)*(U92073) and *Isa*(HM990671) gene, and both isolates were sensitive to erythromycin and resistant to clindamycin. Four isolates harboring the genes *mre(A)*(U92073), *mef(A)*(U83667), and *msr*(AF274302) were sensitive to both erythromycin and clindamycin. One isolate (171-2015) harbored the four genes *mre(A)*(U92073), *erm(A)*(AF002716), *mef(A)*(AJ971089), *msr*(AF274302), and was resistant against both erythromycin and clindamycin (**Table 2**).

**Supplementary Table 2** presents specific antimicrobial susceptibility data for individual isolates.

## Discussion

Serotype distribution of invasive GBS isolates often include an “eleventh” category designated as GBS non-typeable isolates (Slotved and Hoffmann, 2017). Using molecular based methods it is often possible to define a genotype based on the capsular genes (Kapatai et al., 2017). The epidemiological data for clinical GBS NT isolates from the period, 2005–2018 previously presented indicated that approximately 10% of all received clinical isolates were phenotypic NT isolates (Slotved and Hoffmann, 2020; Slotved and Hoffmann, 2017), similar to observations from other countries (Lamagni et al., 2013; Rosini et al., 2015; Alhhazmi et al., 2016; Björnsdóttir et al., 2016).

The selection of GBS related virulence genes in our study was inspired by other studies (Skov Sørensen et al., 2010; Alhhazmi et al., 2016; Rosa-Fraile and Spellerberg, 2017; Shabayek and Spellerberg, 2018). We found that the 16sRNA sequence, *sodA* gene, and the *cfb* gene described as being GBS species specific all were present in the included isolates (Rosa-Fraile and Spellerberg, 2017) (Streptococcus Laboratory, CDC, https://www.cdc.gov/streplab/groupb-strep/index.html, accessed 28-01-2020). The *infB* gene which is a housekeeping gene and has been described as a useful marker for GBS lineages was also detected by us, and we could confirm the usefulness of this gene as a GBS species marker (Skov Sørensen et al., 2010).

The *hvgA* gene connected to the highly virulent ST17/CC17 clone that enables persistent colonization by GBS and contributes to meningitis in neonates (Alhhazmi et al., 2016; Burcham et al., 2019; Kardos et al., 2019) was not found in our strains, except the serotype III reference strain, which belongs to the highly virulent ST17 clone (**Supplementary Table 1**). Both the *scpB* and *lmb* genes were generally detected together in the majority of the isolates in this study (**Table 1**). The two genes are often detected in GBS isolates from humans, while less frequently from animals (Shabayek and Spellerberg, 2018; Chen, 2019). The *scpB* and *lmb* genes in GBS have been associated to an ability to colonize human mucosal surfaces (Shabayek and Spellerberg, 2018), however in our study we found four clinical isolates without these two genes. In addition, 11 clinical isolates were missing the *scpB* gene (**Table 1**). These genes therefore did not seem to be vital for the infectious capacity of our isolates.

Using WGS on our phenotypic defined GBS NT isolates we were able to uncover a genotype for all of them, except one (306-2017). We were not able to detect any known capsular sequences in this isolate using three different previously described sequence analytical methods for genotyping (Sheppard et al., 2016; Kapatai et al., 2017; Metcalf et al., 2017). Although the isolate belonged to CC1, it did not show any close clonal relationship to other isolates with an identified capsular gene (**Figure 1**). This isolate may therefore have lost capsular sequence, a phenomenon which has been previously described in which a strain was lacking the entire capsular locus (Creti et al., 2012), or the isolate has a new capsular sequence not previously described.

For the genotyping we used capsular gene sequence analysis methods from three different studies (Sheppard et al., 2016; Kapatai et al., 2017; Metcalf et al., 2017). We found a general agreement between the three different genotyping methods, although four of the 35 isolates showed discrepancies between the methods (**Supplementary Table 3**). The advantage of method 1 (Metcalf et al., 2017) is the short-read sequences with exact positive definition for the genotype determination. We did not observe conflicting results between method 1 and method 3 (Sheppard et al., 2016), although method 1 was not able to genotype three of the 35 NT isolates, one of which also expressed a phenotypic serotype (116-2016). Method 2 (Kapatai et al., 2017) uses longer sequences, and while it was not able to genotype one of the isolates it also showed disagreeing genotype compared to methods 1 and 3. Method 3 did not present a sequence for GBS serotype IX and is therefore not optimal for general use in genotyping. In general, all three genotyping methods showed limitations, however a suggestion could be to use method 1 because of the simple sequences and clearly defined parameters for identification of specific genotypes. The remaining NT genotypes that method 1 was not able to define, could be tested additionally with the sequences from method 2 or 3. In the present study, this approach resulted in only one isolate with no known capsular sequence detected.

The general GBS epidemiology in Denmark shows a predominance of serotype III followed by serotypes Ia, and serotype V as the third most common serotype (Slotved and Hoffmann, 2020). The preponderance of NT isolates harboring capsular genes for serotype V observed in this study was also observed in our previous study (Slotved and Hoffmann, 2017). Overall, we observed a similar distribution of capsular genes in the NT group in the two studies (Slotved and Hoffmann, 2017). The only major difference was the appearance of capsular genes for serotype III, which in the present study was observed in only one isolate, while in the previous study it was the second most observed capsular gene among the NT isolates (Slotved and Hoffmann, 2017). Other studies on GBS NT isolates have observed that the majority of their genotypic defined GBS NT isolates were genotype V. It is not clear why the NT isolates are dominated by isolates harboring capsular genes for serotype V, but it has been suggested that certain lineages of GBS genotype V isolates are more competent to generate genetic and antigenic diversity (Ramaswamy et al., 2006).

Several studies have suggested that NT isolates may be a problem for the efficacy of future polysaccharide based vaccines, such as the hexavalent vaccine that include serotypes Ia, Ib, II, III, IV, and V (Buurman et al., 2019). If an isolate is phenotypic non-typeable it can be due to low or no expression of capsule or a capsular structural variant that do not react to any available type specific serum (Ramaswamy et al., 2006; Rosini et al., 2015). The present study shows that except for serotype VI and VII sequences were identified for all other capsular serotypes.

From all the Danish invasive GBS isolates from 2015 to 2017 described in the study by Slotved and Hoffmann (2020), we only detected NT isolates from patients with bacteremia in the age group above 21 years of age and particularly the elderly (**Table 1**). However, other studies have shown that NT isolates also can be detected from children, including children with EOD and LOD (Lamagni et al., 2013; Alhhazmi et al., 2016). In our study NT isolates were only related to patients with bacteremia and not from other specimen sites, however we have not been able to find studies showing if NT isolates are specifically related to bacteremia or to specific age groups.

The MLST data showed that the GBS isolates in this study belong to well-known human-pathogenic clone complexes, such as CC1, CC6-8-10, CC19, CC23, CC130 (Rosini et al., 2015; Sørensen et al., 2019). The hypervirulent CC17 frequently associated with late-onset neonatal disease was only found in our reference strain for GBS serotype III (Bisharat et al., 2004; Rosini et al., 2015). In general we found that the MLST were in accordance with the detected genotypes (**Figure 1**).

Similar to pneumococcus (Li et al., 2016), it has been suggested that the penicillin susceptibility can be predicted by the signature of PBP2x. In this study all isolates were penicillin susceptible, and we were therefore only able to compare phenotypic susceptible isolates with the predicted PBP2x MIC values of ≤0.12 mg/L. All clinical isolates correlated with the predicted MIC value, and could be grouped into three different PBP2x profiles 1, 4, and 5, while one reference isolates belonged to profile 2, supporting the PBP2x typing scheme to detect low-level beta-lactam resistance described by Metcalf et al. (2017).

All the 55 clinical isolates had the *mre(A)* gene known to be linked to macrolide and clindamycin resistance in GBS (Clarebout et al., 2001), however only 10 isolates with the *mre(A)* gene showed phenotypic resistance against erythromycin, eight isolates with the *mre(A)* gene showed phenotypic resistance against clindamycin (**Table 2**, **Supplementary Table 2**), and six isolates with the *mre(A)* gene showed resistance to both erythromycin and clindamycin. The erythromycin and clindamycin resistance genes found in the 55 clinical GBS isolates showed a similar relationship regarding the phenotypic susceptibility as also observed in the study by Metcalf et al. (2017). Two isolates were phenotypic clindamycin resistant while sensitive to erythromycin, and both isolates harbored the *Isa(C)* gene, which has been described as causing clindamycin resistance (Metcalf et al., 2017).

Phenotypic NT defined isolates were only detected in patients in the age group above 21 years with bacteremia, and not in the EOD and LOD patient group. Overall we were able to genotype 34 of 35 of the phenotypic non-typeable isolates and there was a majority of genotype V isolates which did not express their capsular gene. The NT isolates belonged to a variety of well-known human-pathogenic clonal complexes. Because of the sequence simplicity and clear cutoff values, the genotyping method recommended by CDC (Metcalf et al., 2017) seems to be the optimal method for genotyping, however we suggest to also use both method 2 or method 3 for those NT isolates, which cannot be genotyped by method 1.

## Data Availability Statement

The datasets presented in this study can be found in online repositories. The names of the repository/repositories and accession number(s) can be found in the article/**Supplementary Material**.

## Author Contributions

H-CS designed the study, analyzed the data, and drafted the manuscript. SH, IK, and KF analyzed and reviewed the data, contributed to the manuscript, and critically revised the manuscript. All authors contributed to the article and approved the submitted version.

## Conflict of Interest

The authors declare that the research was conducted in the absence of any commercial or financial relationships that could be construed as a potential conflict of interest.

## References

[B1] Alhhazmi A. Tyrrell G. J. (2018). Phenotypic and molecular analysis of nontypeable Group B streptococci: identification of cps2a and hybrid cps2a/cps5 Group B streptococcal capsule gene clusters. Emerg. Microbes Infect. 7, 137. 10.1038/s41426-018-0138-6 30087323 PMC6081472

[B2] Alhhazmi A. Hurteau D. Tyrrell G. J. (2016). Epidemiology of Invasive Group B Streptococcal Disease in Alberta, Canada 2003-2013. J. Clin. Microbiol. 54, JCM.00355–16. 10.1128/JCM.00355-16 PMC4922093 27098960

[B3] Ballard M. S. Schønheyder H. C. Knudsen J. D. Lyytikäinen O. Dryden M. Kennedy K. J.. (2016). The changing epidemiology of group B streptococcus bloodstream infection: A multi-national population-based assessment. Infect. Dis. (Auckl). 48, 386–391. 10.3109/23744235.2015.1131330 26759190

[B4] Bisharat N. Crook D. W. Leigh J. Harding R. M. Ward P. N. Coffey T. J.. (2004). Hyperinvasive neonatal Group B [i]Streptococcus[/i] has arisen from a bovine ancestor. J. Clin. Microbiol. 42, 2161–2167. 10.1128/JCM.42.5.2161-2167.2004 15131184 PMC404684

[B5] Björnsdóttir E. S. Martins E. R. Erlendsdóttir H. Haraldsson G. Melo-Cristino J. Kristinsson K. G.. (2016). Changing epidemiology of group B streptococcal infections among adults in Iceland: 1975-2014. Clin. Microbiol. Infect. 22, 379.e9–379.e16. 10.1016/j.cmi.2015.11.020 26691681

[B6] Brigtsen A. K. Dedi L. Melby K. K. Holberg-Petersen M. Radtke A. Lyng R. V.. (2015). Comparison of PCR and serotyping of Group B Streptococcus in pregnant women: The Oslo GBS-study. J. Microbiol. Methods 108, 31–35. 10.1016/j.mimet.2014.11.001 25447890

[B7] Burcham L. R. Spencer B. L. Keeler L. R. Runft D. L. Patras K. A. Neely M. N.. (2019). Determinants of Group B streptococcal virulence potential amongst vaginal clinical isolates from pregnant women. PloS One 14, 1–16. 10.1371/journal.pone.0226699 PMC6919605 31851721

[B8] Buurman E. T. Timofeyeva Y. Gu J. Kim J. Kodali S. Liu Y.. (2019). A novel hexavalent capsular polysaccharide conjugate vaccine (GBS6) for the prevention of neonatal group B streptococcal infections by maternal immunization. J. Infect. Dis. 10965, 1–11. 10.1093/infdis/jiz062 PMC6548902 30778554

[B9] Chen S. L. (2019). Genomic insights into the distribution and evolution of group B streptococcus. Front. Microbiol. 10, 1447. 10.3389/fmicb.2019.01447 31316488 PMC6611187

[B10] Clarebout G. Villers C. Leclercq R. (2001). Macrolide resistance gene mreA of Streptococcus agalactiae encodes a flavokinase. Antimicrob. Agents Chemother. 45, 2280–2286. 10.1128/AAC.45.8.2280-2286.2001 11451686 PMC90643

[B11] Creti R. Imperi M. Pataracchia M. Alfarone G. Recchia S. Baldassarri L. (2012). Identification and molecular characterization of a S. agalactiae strain lacking the capsular locus. Eur. J. Clin. Microbiol. Infect. Dis. 31, 233–235. 10.1007/s10096-011-1298-7 21614482

[B12] Croucher N. J. Page A. J. Connor T. R. Delaney A. J. Keane J. A. Bentley S. D.. (2015). Rapid phylogenetic analysis of large samples of recombinant bacterial whole genome sequences using Gubbins. Nucleic Acids Res. 43, e15. 10.1093/nar/gku1196 25414349 PMC4330336

[B13] Hedegaard J. Hauge M. Fage-Larsen J. Mortensen K. K. Kilian M. Sperling-Petersen H. U.. (2000). Investigation of the translation-initiation factor IF2 gene, infB, as a tool to study the population structure of Streptococcus agalactiae. Microbiology 146, 1661–1670. 10.1099/00221287-146-7-1661 10878130

[B14] Kapatai G. Patel D. Efstratiou A. Chalker V. J. (2017). Comparison of molecular serotyping approaches of Streptococcus agalactiae from genomic sequences. BMC Genomics 18, 429. 10.1186/s12864-017-3820-5 28571573 PMC5455115

[B15] Kardos S. Tóthpál A. Laub K. Kristóf K. Ostorházi E. Rozgonyi F.. (2019). High prevalence of group B streptococcus ST17 hypervirulent clone among non-pregnant patients from a Hungarian venereology clinic. BMC Infect. Dis. 19, 1–10. 10.1186/s12879-019-4626-7 31779587 PMC6883650

[B16] Lamagni T. L. Keshishian C. Efstratiou A. Guy R. Henderson K. L. Broughton K.. (2013). Emerging trends in the epidemiology of invasive group B streptococcal disease in England and Wales, 1991-2010. Clin. Infect. Dis. 57, 682–688. 10.1093/cid/cit337 23845950

[B17] Lambertsen L. Ekelund K. Skovsted I. C. Liboriussen A. Slotved H. C. (2010). Characterisation of invasive group B streptococci from adults in Denmark 1999 to 2004. Eur. J. Clin. Microbiol. Infect. Dis. 29, 1071–1077. 10.1007/s10096-010-0941-z 20676713

[B18] Lambertsen L. M. Ingels H. Schønheyder H. C. Hoffmann S. (2014). Nationwide laboratory-based surveillance of invasive beta-haemolytic streptococci in Denmark from 2005 to 2011. Clin. Microbiol. Infect. 20, O216–O223. 10.1111/1469-0691.12378 24125634 PMC4232002

[B19] Li Y. Metcalf B. J. Chochua S. Li Z. Gertz R. E. Walker H.. (2016). Penicillin-binding protein transpeptidase signatures for tracking and predicting β-lactam resistance levels in Streptococcus pneumoniae. MBio 7, 1–9. 10.1128/mBio.00756-16 PMC4916381 27302760

[B20] Metcalf B. J. Chochua S. Gertz R. E. Hawkins P. A. Ricaldi J. Li Z.. (2017). Short-read whole genome sequencing for determination of antimicrobial resistance mechanisms and capsular serotypes of current invasive Streptococcus agalactiae recovered in the USA. Clin. Microbiol. Infect. 23, 574.e7–574.e14. 10.1016/j.cmi.2017.02.021 28257899

[B21] Ramaswamy S. V. Ferrieri P. Flores A. E. Paoletti L. C. (2006). Molecular characterization of nontypeable group B streptococcus. J. Clin. Microbiol. 44, 2398–2403. 10.1128/JCM.02236-05 16825355 PMC1489475

[B22] Rosa-Fraile M. Spellerberg B. (2017). Reliable Detection of Group B Streptococcus in the Clinical Laboratory. J. Clin. Microbiol. 55, 2590–2598. 10.1128/JCM.00582-17 28659318 PMC5648696

[B23] Rosini R. Campisi E. De Chiara M. Tettelin H. Rinaudo D. Toniolo C.. (2015). Genomic analysis reveals the molecular basis for capsule loss in the group B Streptococcus population. PloS One 10, e0125985. 10.1371/journal.pone.0125985 25946017 PMC4422693

[B24] Sahl J. W. Lemmer D. Travis J. Schupp J. M. Gillece J. D. Aziz M.. (2016). NASP: an accurate, rapid method for the identification of SNPs in WGS datasets that supports flexible input and output formats. Microb. Genomics 2, e000074. 10.1099/mgen.0.000074 PMC5320593 28348869

[B25] Shabayek S. Spellerberg B. (2018). Group B Streptococcal Colonization, Molecular Characteristics, and Epidemiology. Front. Microbiol. 9, 437. 10.3389/fmicb.2018.00437 29593684 PMC5861770

[B26] Sheppard A. E. Vaughan A. Jones N. Turner P. Turner C. Efstratiou A.. (2016). Capsular Typing Method for Streptococcus agalactiae Using Whole-Genome Sequence Data. J. Clin. Microbiol. 54, 1388–1390. 10.1128/JCM.03142-15 26962081 PMC4844738

[B27] Skov Sørensen U. B. Poulsen K. Ghezzo C. Margarit I. Kilian M. (2010). Emergence and global dissemination of host-specific *Streptococcus agalactiae* clones. MBio 1, 1–9. 10.1128/mBio.00178-10 PMC2932510 20824105

[B28] Slotved H.-C. Hoffmann S. (2017). Evaluation of procedures for typing of group B Streptococcus: a retrospective study. PeerJ 5, e3105. 10.7717/peerj.3105 28321367 PMC5357338

[B29] Slotved H.-C. Hoffmann S. (2020). The Epidemiology of Invasive Group B Streptococcus in Denmark From 2005 to 2018. Front. Public Heal. 8, 40. 10.3389/fpubh.2020.00040 PMC7076979 32211361

[B30] Slotved H.-C. Kong F. Lambertsen L. Sauer S. Gilbert G. L. (2007). Serotype IX, a Proposed New Streptococcus agalactiae Serotype. J. Clin. Microbiol. 45, 2929–2936. 10.1128/JCM.00117-07 17634306 PMC2045254

[B31] Sørensen U. B. S. Klaas I. C. Boes J. Farre M. (2019). The distribution of clones of Streptococcus agalactiae (group B streptococci) among herdspersons and dairy cows demonstrates lack of host specificity for some lineages. Vet. Microbiol. 235, 71–79. 10.1016/j.vetmic.2019.06.008 31282381

[B32] Souvorov A. Agarwala R. Lipman D. J. (2018). SKESA: strategic k-mer extension for scrupulous assemblies. Genome Biol. 19, 153. 10.1186/s13059-018-1540-z 30286803 PMC6172800

[B33] Zankari E. Hasman H. Cosentino S. Vestergaard M. Rasmussen S. Lund O.. (2012). Identification of acquired antimicrobial resistance genes. J. Antimicrob. Chemother. 67, 2640–2644. 10.1093/jac/dks261 22782487 PMC3468078

