# Supplementary material for: Molecular Identification of Invasive Non-typeable Group B Streptococcus Isolates From Denmark (2015 to 2017)
Source: Front Cell Infect Microbiol. 2021 Mar 29;11:571901. doi: 10.3389/fcimb.2021.571901 (PMC8039440; doi:10.3389/fcimb.2021.571901)
Supplement: Supplementary file 1 [file Table_1.docx]

Supplementary tables.

**Article Title:** Molecular Identification Of Invasive Non-typeable Group B *Streptococcus* Isolates from Denmark (2015 to 2017)

**Authors:** Hans-Christian Slotved^1^*, Kurt Fuursted^1^, Ioanna Drakaki Kavalari^1^, Steen Hoffmann^1^.

**Affiliations:**

1. Neisseria and Streptococcus Reference Laboratory, Department of Bacteria, Parasites and Fungi, Statens Serum Institut, Copenhagen, Denmark.

***Corresponding author:** Hans-Christian Slotved, Department of Bacteria, Parasites and Fungi,

Bldg. 47/119, Artillerivej 5, DK-2300 Copenhagen S, Denmark.

Phone: +45 32688422, E-mail: [hcs@ssi.dk](mailto:hcs@ssi.dk)

Supplementary table 1. Phenotypic and molecular characteristics for all 55 isolates and ten reference strains. The following genes were present in all 65 isolates: 16sRNA (AF015927.1), *cfb* (JQ289578), *infB* (AJ003164), *rbfB* (AJ003164), and *sodA* (DQ232566.1).

| **Reference strains** | Initial pheno-typical test^a^ | Second pheno-typical test^b^ | Genotype  (Method 1) | Genotype  (Method 2) | Genotype  (Method 3) | MLST (ST) | Clonal Complex (CC) | *hvgA* gene  (Alhhazmi et al 2016) | *scpB* (SAU56908) | *lmb* (AF062533) |
| --- | --- | --- | --- | --- | --- | --- | --- | --- | --- | --- |
| GBS-ref-BIa  O90 (ATCC 12400) | Ia | ND^d^ | Ia | Ia | Ia | 25 | 23 | Absent | Present | Present |
| GBS-ref-BIb  H36 (NTNC 8187) | Ib | ND | Ib | Ib | Ib | 6 | 6-8-10 | Absent | Present | Present |
| GBS-ref-BII  18 RS 21 (NCTC 11079) | II | ND | II | II | II | 19 | 19 | Absent | Present | Present |
| GBS-ref-BIII  M 781 | III | ND | III | III | III | 17 | 17 | Present | Present | Present |
| GBS-ref-BIV  12351 | IV | ND | IV | IV | IV | 10 | 6-8-10 | Absent | Present | Present |
| GBS-ref-BV  SS 1169 | V | ND | V | V | V | 26 | 26 | Absent | Absent | Absent |
| GBS-ref-BVI  NT6 | VI | ND | VI | VI | VI | 103-3-2-1-2-2-unknown | Singleton | Absent | Present | Present |
| GBS-ref-BVII  7271 | VII | ND | VII | VII | VII | 1 | 1 | Absent | Present | Present |
| GBS-ref-BVIII  130013 (Colindal) | VIII | ND | VIII | VIII | VIII | 1 | 1 | Absent | Present | Present |
| GBS-ref-BIX  7214 | IX | ND | IX | IX | Sequence not included^c^ | 8-4-1-5-3-4-unknown | 130 | Absent | Present | Present |
| **Strains from 2017** |  |  |  |  |  |  |  |  |  |  |
| 15-2017 | NT^e^ | NT | VIII | VIII | VIII | 1 | 1 | Absent | Present | Present |
| 63-2017 | NT | Ia | Ia | Ia | Ia | 23 | 23 | Absent | Present | Present |
| 69-2017 | NT | Ia | Ia | Ia | Ia | 24 | 23 | Absent | Present | Present |
| 93-2017 | NT | Ia | Ia | Ia | Ia | 23 | 23 | Absent | Present | Present |
| 182-2017 | NT | NT | Ib | Ib | Ib | 10 | 6-8-10 | Absent | Present | Present |
| 286-2017 | NT | NT | V | V | V | 1 | 1 | Absent | Absent | Absent |
| 292-2017 | NT | NT | Ia | Ia | Ia | 88 | 23 | Absent | Present | Present |
| 306-2017 | NT | NT | Absent | Absent | Absent | 1-1-1-1-2-2-unknown | 1 | Absent | Present | Present |
| 314-2017 | NT | Ia | Ia | Ia | Ia | 23 | 23 | Absent | Present | Present |
| 333-2017 | NT | Ia | Ia | Ia | Ia | 498 | 23 | Absent | Present | Present |
| 365-2017 | NT | NT | Ia | Ia | Ia | 498 | 23 | Absent | Present | Present |
| 405-2017 | NT | NT | Ib | Ib | Ib | 9 | 6-8-10 | Absent | Present | Present |
| 429-2017 | NT | Ia | Ia | Ia | Ia | 23 | 23 | Absent | Present | Present |
| 455-2017 | NT | Ia | Ia | Ia | Ia | 23 | 23 | Absent | Present | Present |
| 466-2017 | NT | NT | Ib | Ib | Ib | 10 | 6-8-10 | Absent | Present | Present |
| 491-2017 | NT | Ia | Ia | Ia | Ia | 23 | 23 | Absent | Present | Present |
| 497-2017 | NT | IX | IX | IX | Sequence not included^c^ | 130 | 130 | Absent | Present | Present |
| 521-2017 | NT | NT | II | II | II | 12 | 6-8-10 | Absent | Present | Present |
| 527-2017 | NT | NT | Ib | Ib | Ib | 1-4-1-3-3-2-unknown | 6-8-10 | Absent | Present | Present |
| 538-2017 | NT | NT | IV | IV | IV | 196 | 196 | Absent | Present | Present |
| 550-2017 | NT | Ia | Ia | Ia | Ia | 486 | 103 | Absent | Absent | Absent |
| 587-2017 | NT | Ia | Ia | Ia | Ia | 23 | 23 | Absent | Present | Present |
| 628-2017 | NT | Ia | Ia | Ia | Ia | 4-6-3-2-1-3-? | 23 | Absent | Present | Present |
| 705-2017 | NT | NT | IX | IX | Sequence not included^c^ | 130 | 130 | Absent | Present | Present |
| 706-2017 | NT | Ia | Ia | Ia | Ia | 23 | 23 | Absent | Present | Present |
| 707-2017 | NT | Ia | Ia | Ia | Ia | 23 | 23 | Absent | Present | Present |
| 712-2017 | NT | NT | V | V | V | 1 | 1 | Absent | Absent | Present |
| **Strains from 2016** |  |  |  |  |  |  |  |  |  |  |
| 40-2016 | NT | V | V | V | V | 1 | 1 | Absent | Absent | Absent |
| 116-2016 | NT | Ia | Absent | Ia | Ia | 23 | 23 | Absent | Present | Present |
| 129-2016 | NT | NT | Ib | Ib | Ib | 10 | 6-8-10 | Absent | Present | Present |
| 145-2016 | NT | NT | IV | IV | IV | 196 | 196 | Absent | Present | Present |
| 168-2016 | NT | NT | V | V | V | 1 | 1 | Absent | Absent | Present |
| 195-2016 | NT | NT | III | III | III | 19 | 19 | Absent | Absent | Absent |
| 196-2016 | NT | NT | V | V | V | 1 | 1 | Absent | Absent | Present |
| 197-2016 | NT | NT | Ia | Ia | Ia | 144 | 23 | Absent | Present | Present |
| 228-2016 | NT | NT | V | V | V | 1 | 1 | Absent | Absent | Present |
| 246-2016 | NT | NT | V | V | V | 1 | 1 | Absent | Absent | Present |
| 267-2016 | NT | NT | Ib | Ib | Ib | 12 | 6-8-10 | Absent | Present | Present |
| 319-2016 | NT | NT | V | V | V | 1 | 1 | Absent | Absent | Present |
| 327-2016 | NT | VII | VII | VII | VII | 1-2-1-1-2-2-unknown | 1 | Absent | Present | Present |
| 348-2016 | NT | NT | Ia | Ia | Ia | 88 | 23 | Absent | Present | Present |
| 465-2016 | NT | NT | Absent | Ia | Ia | 4 | 1 | Absent | Present | Present |
| 549-2016 | NT | IX | IX | IX | Sequence not included^c^ | 130 | 130 | Absent | Present | Present |
| 578-2016 | NT | NT | V | V | V | 1 | 1 | Absent | Absent | Present |
| 653-2016 | NT | NT | II | II | II | 10 | 6-8-10 | Absent | Present | Present |
| **Strains from 2015** |  |  |  |  |  |  |  |  |  |  |
| 21-2015 | NT | NT | IX | IX | Sequence not included^c^ | 130 | 130 | Absent | Present | Present |
| 59-2015 | NT | NT | V | V | V | 1 | 1 | Absent | Absent | Present |
| 96-2015 | NT | NT | III | Ia | III | 19 | 19 | Absent | Present | Present |
| 171-2015 | NT | NT | V | V | V | 19 | 19 | Absent | Present | Present |
| 233-2015 | NT | V | V | V | V | 1 | 1 | Absent | Absent | Present |
| 360-2015 | NT | NT | II | II | II | 28 | 19 | Absent | Present | Present |
| 362-2015 | NT | NT | V | V | V | 7 | 6-8-10 | Absent | Present | Present |
| 405-2015 | NT | Ia | Ia | Ia | Ia | 23 | 23 | Absent | Present | Present |
| 480-2015 | NT | NT | V | V | V | 1 | 1 | Absent | Absent | Present |
| 600-2015 | NT | NT | V | V | V | 1 | 1 | Absent | Absent | Present |

1. Phenotypic results from when the 55 isolates were initially identified and selected.
2. Phenotypic results from retesting the 55 isolates.
3. Method 3 do not present a sequence for genotype IX.
4. Not done (ND).
5. Non-typeable (NT)
