# Supplementary material for: Molecular Identification of Invasive Non-typeable Group B Streptococcus Isolates From Denmark (2015 to 2017)
Source: Front Cell Infect Microbiol. 2021 Mar 29;11:571901. doi: 10.3389/fcimb.2021.571901 (PMC8039440; doi:10.3389/fcimb.2021.571901)
Supplement: Supplementary file 2 [file Table_2.docx]

Supplementary tables.

**Article Title:** Molecular Identification Of Invasive Non-typeable Group B *Streptococcus* Isolates from Denmark (2015 to 2017)

**Authors:** Hans-Christian Slotved^1^*, Kurt Fuursted^1^, Ioanna Drakaki Kavalari^1^, Steen Hoffmann^1^.

**Affiliations:**

1. Neisseria and Streptococcus Reference Laboratory, Department of Bacteria, Parasites and Fungi, Statens Serum Institut, Copenhagen, Denmark.

***Corresponding author:** Hans-Christian Slotved, Department of Bacteria, Parasites and Fungi,

Bldg. 47/119, Artillerivej 5, DK-2300 Copenhagen S, Denmark.

Phone: +45 32688422, E-mail: [hcs@ssi.dk](mailto:hcs@ssi.dk)

Supplementary table 2. Genes related to antimicrobial susceptibility.

| **Reference strains** | Penicilin G  S ≥ 18mm^a^  (1 unit)^b^ | PBP2x  (Metcalf et al 2017) | MLS - Macrolide, lincosamide and streptogramin B (ResFinder-3.1) | Chloramphenicol (phenicol)  (Resfinder-3.1) | Erythromycin R < 21mm^a^ (15µg) / MIC R > 0.5^c^.  (text in red represent resistant isolates) | Clindamycin R <17mm^a^ (2µg) / MIC R > 0.5^c^  (text in red represent resistant isolates) |
| --- | --- | --- | --- | --- | --- | --- |
| GBS-ref-BIa  O90 (ATCC 12400) | Not done | 5 | mre(A)(U92073) | No resistance related gene detected | Not done | Not done |
| GBS-ref-BIb  H36 (NTNC 8187) | Not done | 1 | mre(A)(U92073) | No resistance related gene detected | Not done | Not done |
| GBS-ref-BII  18 RS 21 (NCTC 11079) | Not done | 1 | mre(A)(U92073) | No resistance related gene detected | Not done | Not done |
| GBS-ref-BIII  M 781 | Not done | 2 | mre(A)(U92073) | No resistance related gene detected | Not done | Not done |
| GBS-ref-BIV  12351 | Not done | 1 | mre(A)(U92073) | No resistance related gene detected | Not done | Not done |
| GBS-ref-BV  SS 1169 | Not done | 4 | mre(A)(U92073) | No resistance related gene detected | Not done | Not done |
| GBS-ref-BVI  NT6 | Not done | 1 | mre(A)(U92073) + Isa(C)(HM990671) Macrolide res (Phenotype) | No resistance related gene detected | Not done | Not done |
| GBS-ref-BVII  7271 | Not done | 1 | mre(A)(U92073) | No resistance related gene detected | Not done | Not done |
| GBS-ref-BVIII  130013 (Colindal) | Not done | 1 | mre(A)(U92073) | No resistance related gene detected | Not done | Not done |
| GBS-ref-BIX  7214 | Not done | 4 | mre(A)(U92073) | No resistance related gene detected | Not done | Not done |
| **Strains from 2017** | Penicilin G  S ≥ 18mm^a^  (1 unit)^b^ | PBP2x  (Metcalf et al 2017) | MLS - Macrolide, lincosamide and streptogramin B (ResFinder-3.1) | Chloramphenicol (phenicol)  (Resfinder-3.1) | Erythromycin R < 21mm^a^ (15µg) / MIC R > 0.5^c^.  (text in red represent resistant isolates) | Clindamycin R <17mm^a^ (2µg) / MIC R > 0.5^c^  (text in red represent resistant isolates) |
| 15-2017 | 25 | 1 | mre(A)(U92073) | No resistance related gene detected | 0/256 | 0/256 |
| 63-2017 | 24 | 5 | mre(A)(U92073) | No resistance related gene detected | 28 | 23 |
| 69-2017 | 23 | 5 | mre(A)(U92073) | No resistance related gene detected | 26 | 22 |
| 93-2017 | 22 | 5 | mre(A)(U92073) | No resistance related gene detected | 24 | 23 |
| 182-2017 | 23 | 1 | mre(A)(U92073) | No resistance related gene detected | 27 | 24 |
| 286-2017 | 19 | 1 | mre(A)(U92073) | No resistance related gene detected | 26 | 23 |
| 292-2017 | 23 | 5 | mre(A)(U92073) | No resistance related gene detected | 27 | 24 |
| 306-2017 | 26 | 1 | mre(A)(U92073) | No resistance related gene detected | 26 | 22 |
| 314-2017 | 21 | 5 | mre(A)(U92073) | No resistance related gene detected | 27 | 23 |
| 333-2017 | 26 | 5 | mre(A)(U92073) + Isa(C)(HM990671) Macrolide res (Phenotype) | No resistance related gene detected | 26 | 15/2 |
| 365-2017 | 20 | 5 | mre(A)(U92073) + Isa(C)(HM990671) Macrolide res (Phenotype) | No resistance related gene detected | 26 | 13/0.75 |
| 405-2017 | 23 | 1 | mre(A)(U92073) | No resistance related gene detected | 26 | 23 |
| 429-2017 | 23 | 5 | mre(A)(U92073)+mef(A) (U83667) Macrolide res (Phenotype)+msr(D) (AF274302) Macrolide, Lincosamide and Streptogramin B res (Phenotype) | No resistance related gene detected | 17/8 | 25 |
| 455-2017 | 23 | 5 | mre(A)(U92073) | No resistance related gene detected | 26 | 23 |
| 466-2017 | 23 | 1 | mre(A)(U92073) | No resistance related gene detected | 25 | 22 |
| 491-2017 | 23 | 5 | mre(A)(U92073)+mef(A) (U83667) Macrolide res (Phenotype)+msr(D) (AF274302) Macrolide, Lincosamide and Streptogramin B res (Phenotype) | No resistance related gene detected | 17/8 | 23 |
| 497-2017 | 24 | 4 | mre(A)(U92073) | No resistance related gene detected | 26 | 23 |
| 521-2017 | 28 | 1 | mre(A)(U92073) | No resistance related gene detected | 28 | 26 |
| 527-2017 | 24 | 1 | mre(A)(U92073) | No resistance related gene detected | 26 | 23 |
| 538-2017 | 23 | 1 | mre(A)(U92073) | No resistance related gene detected | 25 | 21 |
| 550-2017 | 24 | 4 | mre(A)(U92073) | No resistance related gene detected | 29 | 25 |
| 587-2017 | 26 | 5 | mre(A)(U92073) | No resistance related gene detected | 28 | 26 |
| 628-2017 | 21 | 5 | mre(A)(U92073) | No resistance related gene detected | 26 | 24 |
| 705-2017 | 24 | 4 | mre(A)(U92073) | No resistance related gene detected | 29 | 24 |
| 706-2017 | 26 | 5 | mre(A)(U92073)+mef(A) (U83667) Macrolide res (Phenotype)+msr(D) (AF274302) Macrolide, Lincosamide and Streptogramin B res (Phenotype) | No resistance related gene detected | 18/8 | 26 |
| 707-2017 | 24 | 5 | mre(A)(U92073) | No resistance related gene detected | 28 | 23 |
| 712-2017 | 25 | 1 | mre(A)(U92073) | No resistance related gene detected | 26 | 23 |
| **Strains from 2016** | Penicilin G  S ≥ 18mm^a^  (1 unit)^b^ | PBP2x  (Metcalf et al 2017) | MLS - Macrolide, lincosamide and streptogramin B (ResFinder-3.1) | Chloramphenicol (phenicol)  (Resfinder-3.1) | Erythromycin R < 21mm^a^ (15µg) / MIC R > 0.5^c^.  (text in red represent resistant isolates) | Clindamycin R <17mm^a^ (2µg) / MIC R > 0.5^c^  (text in red represent resistant isolates) |
| 40-2016 | 27 | 1 | mre(A)(U92073) | No resistance related gene detected | 25 | 23 |
| 116-2016 | 23 | 5 | mre(A)(U92073) | No resistance related gene detected | 27 | 23 |
| 129-2016 | 24 | 1 | mre(A)(U92073) | No resistance related gene detected | 28 | 24 |
| 145-2016 | 23 | 1 | mre(A)(U92073) | No resistance related gene detected | 27 | 23 |
| 168-2016 | 21 | 1 | mre(A)(U92073)+erm(B) (U86375) Macrolide res (Phenotype) | No resistance related gene detected | 0/256 | 0/256 |
| 195-2016 | 21 | 1 | mre(A)(U92073) | No resistance related gene detected | 27 | 25 |
| 196-2016 | 21 | 1 | mre(A)(U92073)+erm(B) (U86375) Macrolide res (Phenotype) | No resistance related gene detected | 0/256 | 0/256 |
| 197-2016 | 29 | 5 | mre(A)(U92073) | No resistance related gene detected | 27 | 24 |
| 228-2016 | 26 | 1 | mre(A)(U92073) | No resistance related gene detected | 28 | 23 |
| 246-2016 | 20 | 1 | mre(A)(U92073) | No resistance related gene detected | 25 | 21 |
| 267-2016 | 22 | 1 | mre(A)(U92073) | No resistance related gene detected | 25 | 21 |
| 319-2016 | 24 | 1 | mre(A)(U92073) | No resistance related gene detected | 26 | 21 |
| 327-2016 | 23 | 1 | mre(A)(U92073) | No resistance related gene detected | 24 | 21 |
| 348-2016 | 23 | 5 | mre(A)(U92073) | No resistance related gene detected | 26 | 24 |
| 465-2016 | 21 | 4 | mre(A)(U92073) | No resistance related gene detected | 24 | 21 |
| 549-2016 | 26 | 4 | mre(A)(U92073) | No resistance related gene detected | 28 | 23 |
| 578-2016 | 26 | 1 | mre(A)(U92073) | No resistance related gene detected | 25 | 22 |
| 653-2016 | 27 | 1 | mre(A)(U92073) | No resistance related gene detected | 27 | 25 |
| **Strains from 2015** | Penicilin G  S ≥ 18mm^a^  (1 unit)^b^ | PBP2x  (Metcalf et al 2017) | MLS - Macrolide, lincosamide and streptogramin B (ResFinder-3.1) | Chloramphenicol (phenicol)  (Resfinder-3.1)1) | Erythromycin R < 21mm^a^ (15µg) / MIC R > 0.5^c^.  (text in red represent resistant isolates) | Clindamycin R <17mm^a^ (2µg) / MIC R > 0.5^c^  (text in red represent resistant isolates) |
| 21-2015 | 23 | 4 | mre(A)(U92073) | No resistance related gene detected | 25 | 23 |
| 59-2015 | 22 | 1 | mre(A)(U92073) | No resistance related gene detected | 26 | 22 |
| 96-2015 | 22 | 1 | mre(A)(U92073) | No resistance related gene detected | 26 | 22 |
| 171-2015 | 22 | 1 | mre(A)(U92073) + erm(A) (AF002716) Macrolide res (Phenotype) + mef(A) (AJ971089) Macrolide res (Phenotype) + msr(D) (AF274302) Macrolide, Lincosamide and Streptogramin B res (Phenotype) | catQ (M55620) Chloramphenicol resistance (Phenotype) | 0/8 | 23 (D-form) |
| 233-2015 | 20 | 1 | mre(A)(U92073) | No resistance related gene detected | 26 | 21 |
| 360-2015 | 23 | 1 | mre(A)(U92073) | No resistance related gene detected | 25 | 21 |
| 362-2015 | 20 | 1 | mre(A)(U92073) | No resistance related gene detected | 24 | 21 |
| 405-2015 | 25 | 5 | mre(A)(U92073) + mef(A) (U83667) Macrolide res (Phenotype) + msr(D) (AF274302) Macrolide, Lincosamide and Streptogramin B res (Phenotype) | No resistance related gene detected | 17/12 | 22 |
| 480-2015 | 22 | 1 | mre(A)(U92073)+erm(B) (U86375) Macrolide res (Phenotype) | No resistance related gene detected | 0/256 | 0/256 |
| 600-2015 | 23 | 1 | mre(A)(U92073)+erm(B) (U86375) Macrolide res (Phenotype) | No resistance related gene detected | 0/256 | 0/256 |

^a^ Inhibition zone diameter (mm)

^b^ All isolates were tested for penicillin susceptibility (penicillin G 1µg discs) according to recommendations by EUCAST from 2015 - 2017 (EUCAST Clinical Breakpoint table v 5.0, table v 6.0, and table v 7.1) (www.eucast.org/clinical_breakpoints).

^c^ The isolates that were resistant according to the inhibition zone diameter found with the disc diffusion test were further tested by determination of MIC using Etest® (bioMérieux, Denmark) in accordance with the recommendations by EUCAST from 2015 - 2017 (EUCAST Clinical Breakpoint table v 5.0, table v 6.0, and table v 7.1) ([www.eucast.org/clinical_breakpoints](http://www.eucast.org/clinical_breakpoints)).
