# Supplementary material for: Molecular Identification of Invasive Non-typeable Group B Streptococcus Isolates From Denmark (2015 to 2017)
Source: Front Cell Infect Microbiol. 2021 Mar 29;11:571901. doi: 10.3389/fcimb.2021.571901 (PMC8039440; doi:10.3389/fcimb.2021.571901)
Supplement: Supplementary file 3 [file Table_3.docx]

Supplementary tables.

**Article Title:** Molecular Identification Of Invasive Non-typeable Group B *Streptococcus* Isolates from Denmark (2015 to 2017)

**Authors:** Hans-Christian Slotved^1^*, Kurt Fuursted^1^, Ioanna Drakaki Kavalari^1^, Steen Hoffmann^1^.

**Affiliations:**

1. Neisseria and Streptococcus Reference Laboratory, Department of Bacteria, Parasites and Fungi, Statens Serum Institut, Copenhagen, Denmark.

***Corresponding author:** Hans-Christian Slotved, Department of Bacteria, Parasites and Fungi,

Bldg. 47/119, Artillerivej 5, DK-2300 Copenhagen S, Denmark.

Phone: +45 32688422, E-mail: [hcs@ssi.dk](mailto:hcs@ssi.dk)

Supplementary table 3. Four isolates showing either no capsule gene or showing disagreeing results between the three genotyping methods.

| **Reference strains** | Initial phenotypic test | Second phenotypic test | Method 1  (Metcalf et al 2017) | Method 2  (Kapatai et al 2017) | Method 3  (Sheppard et al 2016) | MLST (ST) | Clonal Complex (CC) |
| --- | --- | --- | --- | --- | --- | --- | --- |
| 306-2017 | NT^a^ | NT | Absent^b^ | Absent | Absent | 1-1-1-1-2-2-unknown | 1 |
| 116-2016 | NT | Ia | Absent | Ia | Ia | 23 | 23 |
| 465-2016 | NT | NT | Absent | Ia | Ia | 4 | 1 |
| 96-2015 | NT | NT | III | Ia | III | 19 | 19 |

^a^Non-typeable (NT)

^b^ Capsule gene not identified
