# Supplementary material for: Molecular Identification of Invasive Non-typeable Group B Streptococcus Isolates From Denmark (2015 to 2017)
Source: Front Cell Infect Microbiol. 2021 Mar 29;11:571901. doi: 10.3389/fcimb.2021.571901 (PMC8039440; doi:10.3389/fcimb.2021.571901)
Supplement: Supplementary file 4 [file Table_4.docx]

Supplementary tables.

**Article Title:** Molecular Identification Of Invasive Non-typeable Group B *Streptococcus* Isolates from Denmark (2015 to 2017)

**Authors:** Hans-Christian Slotved^1^*, Kurt Fuursted^1^, Ioanna Drakaki Kavalari^1^, Steen Hoffmann^1^.

**Affiliations:**

1. Neisseria and Streptococcus Reference Laboratory, Department of Bacteria, Parasites and Fungi, Statens Serum Institut, Copenhagen, Denmark.

***Corresponding author:** Hans-Christian Slotved, Department of Bacteria, Parasites and Fungi,

Bldg. 47/119, Artillerivej 5, DK-2300 Copenhagen S, Denmark.

Phone: +45 32688422, E-mail: [hcs@ssi.dk](mailto:hcs@ssi.dk)

Supplementary table 4. Results of genotyping of 35 phenotypic non-typeable GBS isolates from 2015-2017. The genotyping is based on the sequence analysis described by method 1.

|  | Ia | Ib | II | III | IV | V | VI | VII | VIII | IX | No capsule gene | Total number of phenotypic NT defined isolates |
| --- | --- | --- | --- | --- | --- | --- | --- | --- | --- | --- | --- | --- |
| 2015 | 0 | 0 | 1 | 1* | 0 | 5 | 0 | 0 | 0 | 1 | 0 | 8 |
| 2016 | 3* | 2 | 1 | 1 | 1 | 6 | 0 | 0 | 0 | 0 | 0 | 14 |
| 2017 | 2 | 4 | 1 | 0 | 1 | 2 | 0 | 0 | 1 | 1 | 1 | 13 |
| Total | 6 | 6 | 3 | 1 | 2 | 13 | 0 | 0 | 1 | 2 | 1 | 35 |

*Disagreeing genotyping results between method 1, method 2 or method 3. All other typing results were identical between all three methods. For data on the disagreeing genotyping results between method 1, method 2 or method 3, see Supplementary table 3.
